# Electrochemotherapy with intravenous, intratumoral, or combined administration of bleomycin in the treatment of colorectal hepatic metastases in a rat model

**DOI:** 10.1038/s41598-024-67878-x

**Published:** 2024-07-29

**Authors:** Antonios E. Spiliotis, Sebastian Holländer, Gudrun Wagenpfeil, Robert Eisele, Spyridon Nika, Orestis Mallis Kyriakides, Matthias W. Laschke, Michael D. Menger, Matthias Glanemann, Gereon Gäbelein

**Affiliations:** 1 Institute for Clinical and Experimental Surgery, Saarland University, 66421 Homburg, Germany; 2 Department of Surgery, Charité Universitätsmedizin Berlin, Campus Charité Mitte, Campus Virchow Klinikum, 13353 Berlin, Germany; 3 Department of General Surgery, Vascular-, Visceral- and Pediatric Surgery, Saarland University Medical Center, 66421 Homburg, Germany; 4 Saarland University Medical Center, Institute for Medical Biometry, Epidemiology and Medical Informatics, 66421 Homburg, Germany; 5 Department of Urology and Pediatric Urology, Saarland University Medical Center, 66421 Homburg, Germany

**Keywords:** Electrochemotherapy, Bleomycin, Drug administration routes, Liver neoplasms, Rats, Liver cancer, Cancer therapy

## Abstract

Electrochemotherapy (ECT) combines the reversible electroporation (rEP) with intravenous (i.v.) or intratumoral (i.t.) administration of chemotherapeutic drugs. We conducted this study to compare the efficacy of i.v., i.t., and i.v. + i.t. injection of bleomycin (BLM) in ECT treatment of colorectal hepatic metastases in a rat model. WAG/Rij rats were randomized into three groups and underwent ECT with i.v., i.t., or i.v. + i.t. injection of BLM. Tumor volumes and oxygenation were measured by means of ultrasound and photoacoustic imaging. Moreover, liver and tumor tissue were analyzed by histology and immunohistochemistry. The i.v. and i.v. + i.t. groups exhibited a 44.0% and 46.6% reduction in oxygen saturation of the tumor tissue when compared to pretreatment values, whereas the i.t. group only showed a reduction of 35.2%. The extent of tumor tissue necrosis did not statistically differ between the groups. However, the i.t. group showed a tendency towards a lower necrosis rate. Cell proliferation, apoptotic cell death, vascularization, and immune cell infiltration were comparable in the treated tumors of the three groups. ECT with i.v. administration of BLM should be preferred in clinical practice, as the combined i.v. + i.t. therapy did not show superior oncological outcomes in the present study.

## Introduction

Electrochemotherapy (ECT) represents a nonthermal ablative procedure that combines the administration of chemotherapeutic drugs with well-dosed electric pulses for cell membrane reversible electroporation (rEP). Through rEP, a transmembrane voltage is induced on the cellular membrane that exceeds a certain threshold value, causing transient formation of hydrophilic nanopores and increased cellular permeability^1,2^. In ECT, the enhanced cellular permeability facilitates the transportation of otherwise poorly penetrating chemotherapeutic drugs into tumor cells, increasing their cytotoxicity and inducing cell death^1,3^.

ECT has become increasingly established amongst the treatment options for cutaneous and subcutaneous neoplastic lesions with response rates of up to 86%^3–5^. These beneficial outcomes have increased the interest in utilizing this method also for hepatic tumors. Currently, several ablation therapies are indicated in the treatment of hepatic cancer as an alternative to surgical resection and liver transplantation, mainly in patients with unresectable cancer or in selected cases with resectable disease^6–8^. Radiofrequency and microwave ablation constitute the most commonly used ablation therapies with the highest efficacy^6,7,9,10^. However, their utilization is not indicated for tumors adjacent to major hepatic vessels or bile ducts due to the heat-sink effect and for tumors in the vicinity of other organs due to the risk of thermal injuries^6–8,11,12^.

Current evidence on the role of ECT in the treatment of liver cancer is limited. In fact, only retrospective studies with a small number of patients have been conducted to evaluate the efficacy of ECT in liver tumors^13^. Moreover, the effects of different administration routes of the chemotherapeutic drug have not been systematically analyzed so far.

In ECT, the chemotherapeutic drug can be administered intravenously (i.v.) or through a direct intratumoral (i.t.) injection^14^. In the therapy of skin cancer, small and single tumors can be treated effectively with i.t. drug administration, whereas for the treatment of multiple and large tumors i.v. therapy leads to better oncological results^14^.

Based on these findings, we herein evaluated the effectiveness of i.v., i.t., and i.v. + i.t. administration of the chemotherapeutic drug bleomycin (BLM) during ECT in a rat liver metastasis model.

## Materials and methods

### Animals

Twenty-four WAG/Rij rats of both gender (males: *n* = 12, females: *n* = 12) with a body weight of 241.0 ± 14.9 g and an age of 39.0 ± 0.6 weeks were used for the experiments (Institute for Clinical and Experimental Surgery, Saarland University, Homburg, Germany). The animals were housed in groups in a temperature- and humidity-controlled 12 h light/dark cycle environment with free access to water and standard laboratory chow (Altromin, Lage, Germany).

The study was performed in accordance with the European legislation on the protection of animals (Directive 2010/63/EU) and the National Institutes of Health guidelines for the Care and Use of Laboratory Animals^15^. All experiments were authorized by the local governmental animal protection committee (Landesamt für Verbraucherschutz, Saarbrücken, Germany; permission number: 21/2019). Authors complied with the ARRIVE guidelines^16^.

### Experimental protocol

The rats were randomized into three groups. Each group consisted of 4 males and 4 females. All animals underwent laparotomy with tumor cell injection in the left liver lobe (day 0). On day 8, relaparotomy was performed and the newly developing tumors were analyzed by means of ultrasound and photoacoustic imaging. Thereafter, the animals were treated as follows: (i) ECT with i.v. administration of BLM (i.v. group), ii) ECT with i.t. injection of BLM (i.t. group), iii) or ECT with combined i.v. + i.t. administration of BLM (i.v. + i.t. group). BLM was used in the experiments, as it showed the highest potentiation of cytotoxicity by rEP (up to 1,000 times) when compared to other chemotherapeutic drugs^1,2,17^.

Five days following the treatment (day 13), the animals underwent relaparotomy for the final ultrasound and photoacoustic imaging of the tumors and for the collection of venous blood samples. Subsequently, the animals were sacrificed by an i.v. overdose of sodium pentobarbital and samples of the left liver lobe, including tumor and normal hepatic tissue, were harvested for further histological and immunohistochemical analyses. The body weight of the animals was measured on days 0, 8, and 13 to assess eventual weight loss due to the treatment.

### Tumor cell injection

CC531 rat colon carcinoma cells (CLS, Heidelberg, Germany), which are syngeneic to WAG/Rij rats, were cultured as described in previously published studies^18^. Under isoflurane anesthesia, we conducted a median laparotomy and subcapsular injection of 5 × 10^5^ CC531 cells (in 50 µL phosphate-buffered saline) on the lower surface of the left liver lobe^19^.

### ECT

The animals were anesthetized by isoflurane inhalation and were fixed in supine position on an electronically regulated heating pad. In the i.v. group, the inferior vena cava was exposed after median laparotomy, and BLM (BLEO-cell® 15 mg, STADAPHARM GmbH) was administered as an i.v. bolus injection at a concentration of 4 U/kg body weight with a 30G × 1/2″ needle (BD Microlance™ 3, Becton Dickinson GmbH, Drogheda, Ireland) according to previously published standards^20^. In the i.t. group, the chemotherapeutic agent was directly injected in the center of the tumor at a concentration of 4 U/kg body weight with a 30G × 1/2″ needle^21^. In the i.v. + i.t. group, BLM was injected i.t. (4 U/kg body weight), followed by an i.v. injection at a concentration of 4 U/kg body weight. This dose was utilized in the combined group with the aim to examine if a double dose via two different application routes is significantly associated with better oncological outcomes compared to the monotherapies.

Thereafter, the liver was mobilized and the left liver lobe was exposed to facilitate the electrodes’ insertion. At the timepoint of treatment (on day 8), the tumors exhibited a mean diameter of 5 mm without significant differences between the groups, as measured by ultrasound imaging, and presented as spherically symmetrical or asymmetrical lesions (data not shown). ECT was performed with the Sennex® Tumor System (BIONMED® Technologies GmbH, Saarbrücken, Germany), as described previously^19^. Eight electric pulses of 100 μs duration were applied between the two electrodes 3 min after the BLM injection^22,23^. In the i.v. + i.t. group, rEP was conducted 3 min after the i.t. injection of BLM. According to the European guidelines for the utilization of ECT, the Sennex® Tumor System provides a voltage between the pair of electrodes at 1.000 V, corresponding to an amplitude of 125 V/mm and frequency of 1 Hz^4,24^.

### Ultrasound and photoacoustic imaging

The ultrasound and photoacoustic imaging of the tumors were performed with the Vevo LAZR system (FUJIFILM VisualSonics Inc., Toronto, ON, Canada) and a real-time microvisualization LZ550 linear-array transducer (FUJIFILM VisualSonics Inc.) with a center frequency of 40 MHz. The ultrasound examination was conducted during the laparotomy on day 8 before treatment and on day 13 before sacrificing the animals. The examination was performed according to published standards^19^. OxyHemo-mode photoacoustic images were taken at two wavelengths (750 and 850 nm) with a two-dimensional photoacoustic gain of 42 dB and a hemoglobin threshold of 20 dB. Photoacoustic examination was performed at 750 and 850 nm wavelength illumination to deduce oxygen saturation (SO_2_) from the oxygenated and deoxygenated hemoglobin signal^25^. Furthermore, at these wavelengths any potential effect of edema caused by the ECT treatment on the photoacoustic signal was minimal due to low water absorption^26^. Hemoglobin concentration (HbT) and SO_2_ were calculated in the whole tumor tissue.

### Analysis of blood samples

Venous blood samples were taken via puncture of the subhepatic vena cava on day 13 directly after the ultrasound imaging. The total number of leukocytes (10^9^/L), lymphocytes (10^9^/L), monocytes (10^9^/L), neutrophils (10^9^/L), erythrocytes (10^12^/L), and platelets (10^9^/L) as well as the hemoglobin concentration (g/dL) and hematocrit (%) were assessed by means of a cell counter (VetScan HM5, Firma Scil Animal Care Company GmbH, Viernheim, Germany). Using spectrophotometry, aspartate aminotransferase (ASAT) and alanine aminotransferase (ALAT) serum activities were determined as indicators of hepatocellular injury.

### Histological and immunohistochemical analysis

The histological and immunohistochemical analysis was performed according to our previous published standards^19^. The tumor tissue and the surrounding normal hepatic parenchyma were fixed in 4% phosphate-buffered formalin, embedded in paraffin, and cut into 3-μm thick sections. For the assessment of the necrotic tumor tissue, sections were stained with hematoxylin and eosin^19^.

For the immunohistochemical detection of apoptotic cells, sections were stained with a rabbit polyclonal anti-cleaved caspase-3 antibody (1:100, Cell Signaling Technology, Frankfurt, Germany). For the streptavidin–biotin complex peroxidase staining, a biotinylated anti-rabbit IgG antibody served as secondary antibody (ready-to-use, Abcam, Cambridge, UK)^19^. For the immunohistochemical detection of proliferating cells, sections were stained with a monoclonal mouse-anti-human anti-proliferating cell nuclear antigen (PCNA) antibody (1:100; Dako, Hamburg, Germany). A peroxidase-conjugated goat anti-mouse IgG antibody (1:100; Dianova, Hamburg, Germany) served as secondary antibody^19^. For the immunohistochemical detection of microvessels, sections were stained with a polyclonal rabbit anti-CD31 antibody (1:200; Abcam, Cambridge, UK). A biotinylated goat anti-rabbit IgG antibody (ready-to-use, Abcam, Cambridge, UK) served as secondary antibody^19^. For the immunohistochemical detection of neutrophilic granulocytes, sections were incubated with a rabbit polyclonal anti-myeloperoxidase (MPO) antibody (1:100; Abcam, Cambridge, UK) as primary antibody, followed by a biotinylated goat anti-rabbit IgG antibody (ready-to-use; Abcam, Cambridge, UK). 3-Amino-9-ethylcarbazole (Abcam, Cambridge, UK) or 3.3′-diamino-benzidine were used as chromogens, and Mayer’s hemalum (Merck, Darmstadt, Germany) served as counterstaining^19^.

A BX60 microscope (Olympus, Hamburg, Germany) and the imaging software cellSens Dimension 1.15 (Olympus, Hamburg, Germany) were used for quantitative histological and immunohistochemical analyses. For this purpose, the sections were coded and microscopically analyzed by three independent researchers, who were blinded with respect to the treatment group. Necrotic areas in the tumor tissue were measured as percentage of the whole tumor area on the section with the largest cross-sectional diameter of the tumor^19^. For the assessment of cell proliferation, a semiquantitative index was developed. Accordingly, every specimen was classified into one of five categories based on the percentage of PCNA-positive tumor cells relative to the total number of tumor cells in 10 high power fields (HPF) of non-necrotic tumor tissue (0: ˂ 1%, 1: 1–10%, 2: 11–30%, 3: 31–50%, and 4: ˃ 50% of PCNA-positive cells)^19^. Cleaved caspase-3-positive cells, MPO-positive neutrophilic granulocytes as well as CD31-positive microvessels were counted in 10 randomly selected HPF of non-necrotic tumor tissue (5 HPF in the center and 5 HPF in the periphery of the tumor). Positive cells were given as absolute number per HPF and the microvessel density was given in mm^−2^^19^.

### Statistical analysis

Since sample sizes in all group were lower than 12, we utilized parametric tests in any case. In this way, all values are expressed as mean ± SEM (standard error of the mean for consistency). Differences between the groups were calculated by one-way analysis of variance (ANOVA). We did not account for the issue of multiple post-hoc testing due to the explorative nature of the investigation. The pairwise comparison was performed by a Student’s t test. Overall statistical significance is due to a two-sided significance level of 0.05. Statistical analysis was performed with the use of IBM-SPSS, version 28.0.1.0.

## Results

### Tumor development and general health conditions

All animals developed a hepatic tumor in the left liver lobe on day 8 following tumor cell injection without any signs of peritoneal or extrahepatic metastases. The assessment of body weight at regular intervals revealed a slight but not significant decrease from day 0 to day 13, which did not exceed 10% of the total body weight (data not shown). The animals were not affected systemically by the malignant process and showed normal activity as well as feeding and cleaning habits throughout the observation period.

### Ultrasound and photoacoustic imaging

**Figure 1:**
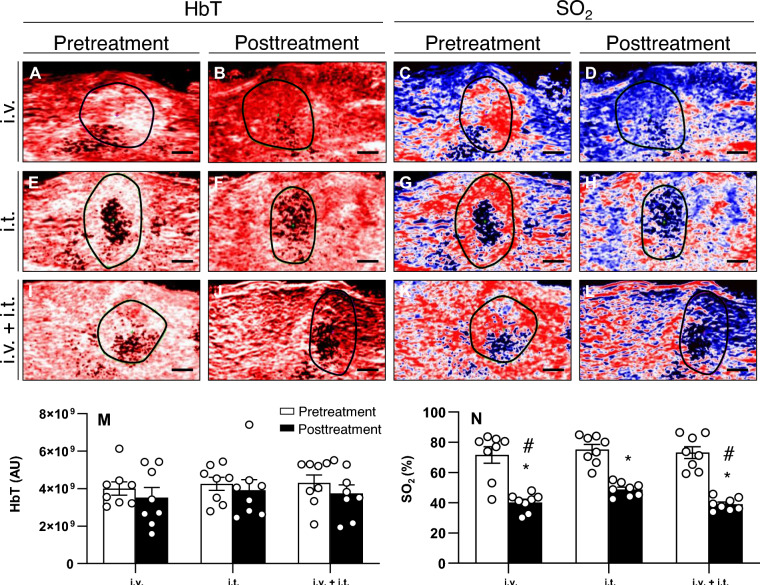
(**A**–**L**) Photoacoustic imaging with pretreatment (on day 8) and posttreatment (on day 13) hemoglobin map and oxygen saturation map in animals treated with ECT and i.v. (**A**–**D**), i.t. (**E**–**H**), or i.v. + i.t. (**I**–**L**) administration of BLM. In the oxygen saturation map, the lowest saturation is dark blue, whereas the highest levels are red. Black lines indicate the tumor outline. Scale bars: 1 mm. M–N) HbT and SO_2_ were measured in the whole tumor area. Data are given as mean ± SEM; *p < 0.05 vs. SO_2_ pretreatment in each group; ^#^*p* < 0.05 vs. SO_2_ posttreatment in i.t. group.

On day 8, ultrasound and photoacoustic examination was conducted to evaluate the pretreatment imaging characteristics of the tumors. At this time point, the tumor volume (i.v.: 39.7 ± 6.4 mm^3^; i.t.: 39.5 ± 3.0 mm^3^; i.v. + i.t.: 46.0 ± 3.4 mm^3^; *p* = 0.527), HbT, and SO_2_ were comparable among the three groups (Fig. 1).

HbT and SO_2_ were additionally assessed five days following the ECT procedure (day 13), whereas the tumor volume could not be exactly measured anymore due to edema formation. The i.v. + i.t. group demonstrated the largest reduction in HbT (13.1%) compared to pretreatment values, whereas in the i.v. and i.t. groups the reduction in HbT was lower with 12.0 and 8.0%, respectively (Fig. 1). Despite the reduction in HbT of the tumor tissue in all groups after ECT, the comparison of pre- and posttreatment values revealed no significant differences after treatment in each group. Furthermore, the analysis of the posttreatment HbT levels revealed no significant differences among the groups.

The i.v. and the i.t. group exhibited a 44.0 and 35.2% reduction in SO_2_ of the tumor area compared to pretreatment values, respectively. The largest reduction in SO_2_ was detected in the i.v. + i.t. group with 46.6%. Finally, the comparison of posttreatment oxygenation levels among the groups revealed a significantly reduced SO_2_ after i.v. (40.1%) and i.v. + i.t. (42.7%) injection when compared to the i.t. treatment (48.8%) on day 13 (Fig. 1).

### Tumor necrosis

The tumors in the three groups demonstrated similar microscopic characteristics in the histological analysis of hematoxylin–eosin–stained sections. Specifically, extensive necrosis was detected in the treated areas, which was surrounded by a fibrotic pseudocapsule consisting of fibroblastic tissue, inflammatory cells (foamy macrophages, lymphocytes, plasma cells, eosinophils, and histiocytes), and proliferating microvessels with red blood cell (RBC) extravasates. A limited number of residual tumor cells in combination with inflammatory cell infiltration was seen in the periphery of the treated area. The fibrotic pseudocapsule was surrounded by regenerative marginal hepatocytes and normal hepatic parenchyma without tumor infiltration. Finally, the wall of vessels and bile ducts adjacent to the ablation zone remained intact in all groups.

**Figure 2:**
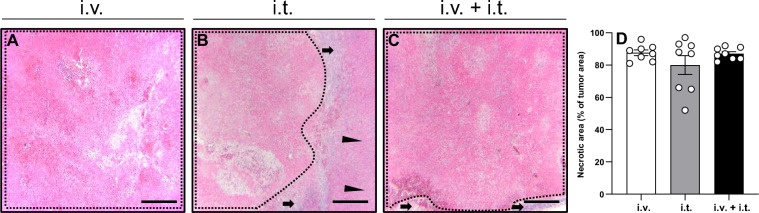
(**A**–**C**) Histological analysis of necrotic cell death (borders marked by dotted line) in animals treated with ECT and i.v. (**A**), i.t. (**B**), or i.v. + i.t. (**C**) administration of BLM. The fibrotic pseudocapsule with residual tumor cells is marked by arrows and normal hepatic tissue by arrowheads. Scale bars: 50 µm. (**D**) Necrotic areas in the tumor tissue were measured as percentage of the whole tumor area. Data are given as mean ± SEM.

The histological analysis revealed a higher rate of tumor necrosis in the groups treated with i.v. or combined i.v. + i.t. administration of BLM when compared to the group treated with i.t. injection of BLM. The i.t. group presented with 79.8% necrosis of the hepatic tumor, whereas the groups treated with i.v. and i.v. + i.t. administration of BLM exhibited necrosis rates of 87.6 and 87.0%, respectively (Fig. 2).

### Apoptotic cell death and tumor cell proliferation

**Figure 3:**
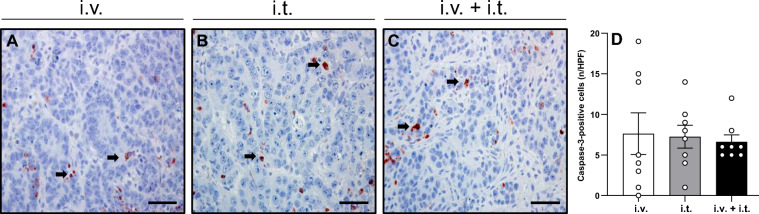
(**A**–**C**) Immunohistochemical analysis of cleaved caspase-3 in animals treated with ECT and i.v. BLM (**A**), i.t. BLM (**B**), or i.v. + i.t. (**C**) administration of BLM. Apoptotic cells (arrows) are stained red. Scale bars: 50 µm. (**D**) The diagram displays the mean number of positive cells in the tumor tissue per HPF. Data are given as mean ± SEM.

**Figure 4:**
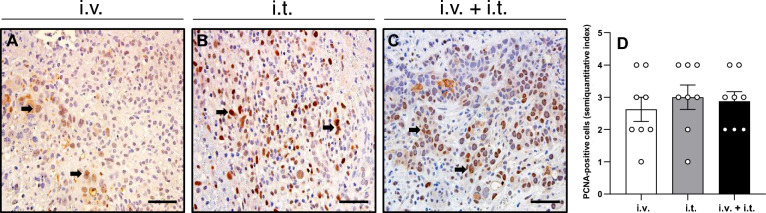
(**A**–**C**) Immunohistochemical sections of PCNA expression in the tumor tissue of animals undergoing ECT with i.v. BLM (**A**), i.t. BLM (**B**), or i.v. + i.t. (**C**) administration of BLM. PCNA-positive cells (arrows) are stained brown. Scale bars: 50 µm. (**D**) The diagram displays the quantitative analysis of PCNA-positive cells (semiquantitative index) in the tumor tissue per HPF. Data are given as mean ± SEM.

A limited number of apoptotic cells was observed in the periphery of the ablation zone among the residual tumor cells and the fibrotic pseudocapsule. The rate of apoptotic cells was similar among the groups (Fig. 3). Moreover, the immunohistochemical analysis of tumor cell proliferation revealed a comparable fraction of PCNA-positive cells in the tumor tissue of all three groups (Fig. 4).

### Tumor vascularization and inflammatory response

**Figure 5:**
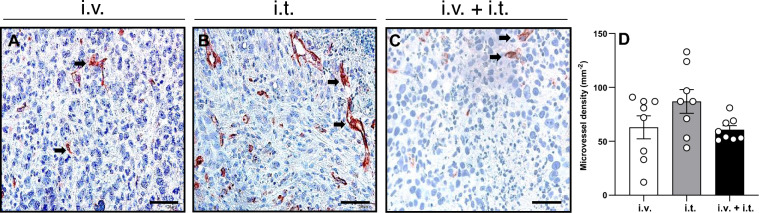
(**A**–**C**) Immunohistochemical analysis of CD31 expression in the tumor tissue of animals treated with ECT and i.v. BLM (**A**), i.t. BLM (**B**), or i.v. + i.t. (**C**) administration of BLM. CD31-positive blood vessels (arrows) are stained red. Scale bars: 50 µm. (**D**) The diagram shows the quantitative analysis of CD31-positive blood vessels in tumor tissues. The microvessel density is given as the number of positive vessels (in 10 HPF) per mm^2^.

The immunohistochemical analysis of CD31-positive microvessels showed that the i.v. and the combined i.v. + i.t. injection of BLM reduced the microvessel density in the tumor tissue by 27.6 and 30.1% when compared to the i.t. injection, respectively (Fig. 5).

**Figure 6:**
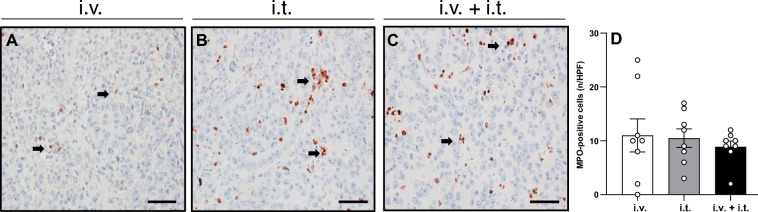
(**A**–**C**) Immunohistochemical analysis of MPO expression in the tumor tissue of animals treated with ECT and i.v. BLM (**A**), i.t. BLM (**B**), or i.v. + i.t. (**C**) administration of 6BLM. MPO-positive cells are stained red (arrows). Scale bars: 50 µm. (**D**) The diagram displays the quantitative analysis of MPO-positive cells in tumor tissue per HPF. Data are given as mean ± SEM.

The immunohistochemical detection of MPO-positive neutrophilic granulocytes revealed a similar number of inflammatory cells in the tumor tissue following ECT treatment in the three groups (Fig. 6).

### Blood sample analysis

The analysis of the white blood cell count revealed physiological values on day 13 in all groups. The animals that underwent i.t. injection of BLM presented a slight but not significant increase of leukocytes and lymphocytes when compared to the other two groups. RBC and platelet counts were within physiological ranges in all groups. Finally, hemoglobin and hematocrit levels were also not affected by the ECT procedure in the three groups (Table 1).

**Table 1:** Blood sample analysis on day 13.

	i.v	i.t	i.v. + i.t
Leukocytes (109/l)	7.5 ± 0.5	8.7 ± 1.2	7.6 ± 0.7
Lymphocytes (109/l)	4.7 ± 0.3	5.6 ± 0.6	4.9 ± 0.5
Monocytes (109/l)	0.5 ± 0.1	0.4 ± 0.1	0.3 ± 0.1
Neutrophils (109/l)	2.3 ± 0.3	2.6 ± 0.7	2.3 ± 0.3
RBC (1012/l)	6.7 ± 0.2	7.4 ± 0.4	7.1 ± 0.2
Platelets (g/dl)	601.9 ± 27.9	639.9 ± 26.8	648.8 ± 30.6
Hemoglobin (g/dl)	13.4 ± 0.4	13.8 ± 0.6	13.8 ± 0.5
Hematocrit (%)	38.4 ± 0.9	41.1 ± 1.5	40.2 ± 1.0
ASAT (U/l)	273.1 ± 28.3	250.5 ± 31.4	252.8 ± 17.3
ALAT (U/l)	170.8 ± 18.2	145.0 ± 24.1	167.8 ± 13.9

Data are given as mean ± SEM.

The analysis of liver enzymes as indicators of hepatocellular injury did not show any relevant differences among the three groups. The liver enzyme values were within physiological ranges on day 13 (Table 1).

### Adverse events

Complications related to electrode insertion, electric pulse delivery or BLM administration were not detected in the present study. The animals showed no clinical signs of a systemic inflammatory response or liver failure at any point during the study.

## Discussion

The beneficial outcomes of ECT in the treatment of cutaneous cancer have increased the interest in using this method also for hepatic tumors, since this non-thermal procedure may be particularly beneficial in the vicinity of high-risk areas, preserving the patency of vessels and bile ducts^27,28^. In a previous study, we could already demonstrate that ECT with i.v. administration of BLM leads to extensive necrosis, reduced vascularization, and decreased oxygenation of the treated tumor tissue^19^. Based on these promising findings, we assessed in the present study the effectiveness of three different administration routes of BLM during ECT in a rat liver metastasis model.

Initially, we proved that the included animals exhibited a similar tumor development and tumor characteristics. In fact, our sonographic and photoacoustic examination on the day of treatment revealed that the tumor volume, tumor SO_2_ and HbT were similar among the studied groups. Consequently, the different treatment approaches were examined in tumors with same biology and comparable volume and vascularization.

Necrosis comprises the most crucial factor in the efficacy assessment of ECT treatment. Regarding this parameter, the histological analysis of the present study revealed no statistically significant difference between the treated groups. However, a remarkably higher rate of necrosis was observed in the groups treated with i.v. or i.v. + i.t. administration of BLM.

The reduced rate of necrosis following i.t. treatment compared to i.v. injection can be explained by the tumor characteristics. CC531 tumors are characterized by a heterogeneous tumor microenvironment with regional variations in perfusion, tissue oxygenation and blood volume^29^. This finding was confirmed by our sonographic and photoacoustic analysis. However, no areas of extensive hypoxia or absence of microvessels were found. Hence, in the i.v. group BLM could reach most of the tumor cells via the microcirculation. In contrast, the less beneficial outcomes after i.t. injection may have been caused by an insufficient interstitial distribution of the chemotherapeutic drug within the tumor tissue. This hypothesis is supported by our histological results, where an increased number of residual tumor cells in combination with inflammatory cell infiltration was particularly detected in the periphery of the tumors after i.t. treatment.

The second factor, which may have caused differences in tumor necrosis, is the ECT-induced alteration of tumor vascularization. It has been proven that the effectiveness of ECT is enhanced through the “vascular disrupting effect” and the “vascular lock effect”^2,3^. According to the “vascular disrupting effect”, the increased cell permeability through rEP is not limited to the tumor cells but extends to the endothelial vascular cells in the treated area. The subsequent increased diffusion of BLM into the endothelial cells leads to cellular damage, occlusion of blood flow, and ischemic death of the tumor cells adjacent to the obstructed blood vessels^30^. In our study, the “vascular disrupting effect” was limited in the i.t. group, since the concentration of BLM in the tumor vessels was lower when compared to i.v. injection. Consequently, we observed lower necrosis rates the i.t. treatment group. Moreover, it is known that electrical stimulation of the precapillary smooth muscle cells causes direct vasoconstriction followed by indirect sympathetically mediated vasoconstriction of the afferent arterioles^2,3,30^. Furthermore, the shape of vascular endothelial cells is modified by the electrical fields, leading to increased vascular resistance and alteration of the endothelial cell-to-cell junctions^2^. Therefore, the ECT-induced hypoperfusion in the treated area further increases the efficacy of i.v. BLM administration, since it is associated with drug entrapment in the tumor tissue and an increased retaining time of the chemotherapeutic agent, limiting the wash-out phenomenon in hypervascularized tissues^2,3,30^.

Of note, the combined i.t. + i.v. administration of BLM resulted in a necrosis and proliferation rate, which was comparable to that in the i.v. group. Hence, the double dose of chemotherapy did obviously not lead to better oncological outcomes. In this context, it should be considered that the pharmacokinetic profile of BLM in the distribution phase after bolus injection is not known in each tumor model. Preliminary data from clinical studies in cutaneous cancer concluded that a complete response in ECT-treated lesions can be achieved with a minimal intracellular concentration of 14–18 ng/g^31^. In our study, we used BLM at a dose of 4 U/kg for i.v. as well as i.t. administration. Our results indicate that this dose was already sufficient in our rat model to reach this minimal intracellular concentration after rEP. Consequently, higher doses of BLM, as used in the i.v. + i.t. group, could not further increase the cytotoxicity of our treatment. This assumption is supported by the results of a preclinical study conducted in a mouse model of subcutaneous colorectal metastases^32^. The authors of this study reported a comparable anti-tumor effectiveness in animals that were treated with 1, 5, or 25 mg/mL BLM.

The mechanism of cell death following ECT remains unclear and studies suggest that the cytotoxic effects of ECT are likely to be cell-specific^33,34^. In our study, we did not detect increased rates of apoptotic cell death following ECT. Therefore it may be concluded that ECT combined with BLM, regardless of the administration route, is not a treatment modality that activates apoptotic signaling pathways to eliminate the development of cancer cells.

In our study, we used photoacoustic imaging to provide a high-resolution 3D tomographic map of oxygen saturation by measuring oxygenated and deoxygenated hemoglobin. Tumor oxygenation was decreased following ECT treatments, which is attributed to the extensive necrosis of the tumor tissue as well as to the ECT vascular effects. The “vascular lock effect” led to decreased SO_2_ and HbT in all groups, inducing vasoconstriction and hypoperfusion of the tumor tissue. In our analysis, the i.v. and i.v. + i.t. groups showed statistically decreased levels of SO_2_ in comparison to the i.t. group, which is interpreted by two factors. Firstly, in the i.t. group, we found the lowest rate of necrosis, which was associated with residual vital tumor tissue and increased tumor vascularization. As an expected result, these tumor regions showed increased SO_2_ and HbT. Secondly, the occluded microvascularization adjacent to the tumor tissue through the “vascular disrupting effect” was more enhanced in the i.v. and i.v. + i.t. groups, leading to decreased SO_2_ and HbT. In agreement with the imaging results, the analysis of CD31-positive microvessels showed a 28–30% reduction in microvessel density after i.v. injection when compared to i.t. injection of BLM.

The immunohistochemical detection of MPO-positive neutrophilic granulocytes revealed a limited number of inflammatory cells in the tumor tissue of all groups following ECT treatment. As previously reported^18^, this result may be attributed to increased necrosis of the malignant cells after ECT, which limits the need of granulocytes to proliferate and to induce cellular death by phagocytosis. In line with this view, other preclinical and clinical studies also reported a limited tissue infiltration of neutrophilic granulocytes after ECT. They rather detected lymphocytes and plasma cells at the margin of the ablated area and within the normal liver parenchyma^35,36^.

Finally, it should be mentioned that the present study has several limitations. Following the 3R principle (replacement, reduction, and refinement), we performed our experiments only in a minimal number of animals, which precluded more statistical analyses of the obtained data. Moreover, our results are limited to the treatment of colorectal hepatic metastases. Hence, additional studies are required to assess the efficacy of ECT in the treatment of tumors with other pathology. Specifically, hepatocellular cancer exhibits an enhanced vascularization when compared to colorectal metastases, which are more fibrotic^37^. This difference in tumor vascularization could lead to an increased diffusion of BLM into hepatocellular tumors and, thus, to an enhanced efficacy of the i.v. treatment.

## Conclusions

Taken together, the present study demonstrates that the combination of i.v. + i.t. administration of BLM does not improve the oncological outcome when compared to i.v. or i.t. administration of the chemotherapeutic drug. Since in clinical practice the i.v. administration of chemotherapeutic drugs is the commonly applied procedure, we therefore recommend to retain the i.v. route as the standard approach in ECT.

## Data Availability

The datasets generated during and/or analysed during the current study are available from the corresponding author on reasonable request.
